# Mitophagy and clear cell renal cell carcinoma: insights from single-cell and spatial transcriptomics analysis

**DOI:** 10.3389/fimmu.2024.1400431

**Published:** 2024-06-27

**Authors:** Lai Jiang, Xing Ren, Jinyan Yang, Haiqing Chen, Shengke Zhang, Xuancheng Zhou, Jinbang Huang, Chenglu Jiang, Yuheng Gu, Jingyi Tang, Guanhu Yang, Hao Chi, Jianhua Qin

**Affiliations:** ^1^ Clinical Medical College, Southwest Medical University, Luzhou, China; ^2^ Department of Oncology, Chongqing General Hospital, Chongqing, China; ^3^ School of Stomatology, Southwest Medical University, Luzhou, China; ^4^ Department of Specialty Medicine, Ohio University, Athens, OH, United States; ^5^ Department of Nephrology, Affiliated Hospital of Southwest Medical University, Luzhou, China; ^6^ Department of Nephrology, Sichuan Clinical Research Center for Nephropathy, The Affiliated Hospital of Southwest Medical University, Luzhou, Sichuan, China

**Keywords:** clear cell renal cell carcinoma, mitophagy, mitochondrial gene defects, multi-omics analysis, metabolic reprogramming, prognostic analysis, non-negative matrix factorization

## Abstract

**Background:**

Clear Cell Renal Cell Carcinoma (ccRCC) is the most common type of kidney cancer, characterized by high heterogeneity and complexity. Recent studies have identified mitochondrial defects and autophagy as key players in the development of ccRCC. This study aims to delve into the changes in mitophagic activity within ccRCC and its impact on the tumor microenvironment, revealing its role in tumor cell metabolism, development, and survival strategies.

**Methods:**

Comprehensive analysis of ccRCC tumor tissues using single cell sequencing and spatial transcriptomics to reveal the role of mitophagy in ccRCC. Mitophagy was determined to be altered among renal clear cells by gene set scoring. Key mitophagy cell populations and key prognostic genes were identified using NMF analysis and survival analysis approaches. The role of UBB in ccRCC was also demonstrated by *in vitro* experiments.

**Results:**

Compared to normal kidney tissue, various cell types within ccRCC tumor tissues exhibited significantly increased levels of mitophagy, especially renal clear cells. Key genes associated with increased mitophagy levels, such as UBC, UBA52, TOMM7, UBB, MAP1LC3B, and CSNK2B, were identified, with their high expression closely linked to poor patient prognosis. Particularly, the ubiquitination process involving the UBB gene was found to be crucial for mitophagy and its quality control.

**Conclusion:**

This study highlights the central role of mitophagy and its regulatory factors in the development of ccRCC, revealing the significance of the UBB gene and its associated ubiquitination process in disease progression.

## 1 Introduction

Renal cancer is a common malignant tumor, with its incidence continuously increasing worldwide (1). Despite some progress in treatment, many mysteries still remain regarding the pathogenesis of renal cancer (2). Clear Cell Renal Cell Carcinoma (ccRCC) is one of the most common types of renal cancer, occupying a major proportion of malignant kidney tumors (3, 4). This cancer typically originates from the epithelial cells of renal tubules and is characterized by high heterogeneity and complexity (5, 6). Compared to other tumor types, the treatment options for ccRCC are relatively limited, making it crucial to deepen our understanding of its pathogenesis for developing more effective treatment plans (7, 8).

Mitochondrial defects refer to structural or functional abnormalities in mitochondria, which can be caused by various factors, including genetic mutations, damage induced by environmental factors, increased oxidative stress, or damage to mitochondrial DNA (mtDNA) (9, 10). These defects often lead to an increased frequency of mitophagy. This is because mitochondrial defects, such as DNA damage, improper protein folding, increased oxidative stress, or insufficient energy production, can impair the normal function of mitochondria (11, 12).

In recent years, increasing evidence has suggested that mitophagy plays a key role in tumors (13, 14). Mitophagy is an intracellular self-degradation process through which cells can remove damaged mitochondria, thereby maintaining mitochondrial health (15). However, when mitophagy is dysregulated, it can lead to mitochondrial dysfunction, abnormal cell metabolism, and cell death (16). The anomalies in mitophagy associated with ccRCC suggest a close link between the two. In ccRCC, abnormalities in mitophagy may be caused by various factors, including changes in the intracellular and extracellular environment, genetic mutations, and dysregulation of regulatory pathways (17). These abnormalities not only affect the survival and proliferation of tumor cells but may also impact tumor development, invasion, and drug resistance (18).

This study aims to explore the connection between ccRCC and mitophagy genes through multi-omics analyses such as single-cell sequencing and spatial transcriptomics, revealing the importance of potential molecular aspects in the progression of renal cancer disease.

## 2 Materials and methods

### 2.1 Source of raw data

The single cell sequencing data of ccRCC used in this study were sourced from the Gene Expression Omnibus (GEO, https://www.ncbi.nlm.nih.gov/geo/) dataset GSE210038, which includes tumor samples from three patients with ccRCC (GSM6415686, GSM6415687, and GSM6415689) and one sample of normal adjacent tissue (GSM6415694). Through the analysis of these single-cell data, the study delves into the heterogeneity differences at the cellular level between renal cell carcinoma and adjacent normal tissues. The spatial transcriptomics data were also obtained from the GEO database (GSE210041), covering sequencing data for two formalin-fixed paraffin-embedded (FFPE) ccRCC tumor samples. This dataset provides a unique perspective for studying the spatial distribution heterogeneity of ccRCC and its surrounding environment. Additionally, RNA sequencing data for ccRCC were downloaded from the UCSC Xena platform (https://xena.ucsc.edu/), originating from the TCGA (The Cancer Genome Atlas) cohort, including sequencing information for 607 samples along with corresponding survival data for survival analysis, thereby enhancing our understanding of prognostic factors for ccRCC. Furthermore, genes related to mitophagy were sourced from the GSEA website (https://www.gsea-msigdb.org/gsea/index.jsp).

### 2.2 Processing of single-cell sequencing data

In this study, we analyzed the single-cell RNA-seq data of ccRCC using the Seurat package (version 4.3.0) in R (19). Through strict quality control, cells with a gene expression range of 200–4000 and mitochondrial gene expression ratio below 20% were selected. After standardization and normalization of the data, important principal components were determined using RunPCA and JackStraw analysis, followed by clustering and visualization with t-SNE to display the similarities and differences among cells. Differential expression analysis was conducted using the FindAllMarkers function, and cell types were annotated in conjunction with the CellMarker database (http://xteam.xbio.top/CellMarker/index.jsp), providing a data foundation for revealing the molecular mechanisms and potential therapeutic targets of ccRCC.

Five gene set scoring methods (AddModuleScore, ssGSEA, AUCell, UCell, singscore) were employed to score mitophagy-related genes in single-cell data. The mitophagy-related genes were obtained from the GSEA website and include 29 genes. The proteins encoded by these genes are involved in various processes including autophagosome formation, the composition of protein kinase CK2, mitochondrial fusion, mitochondrial fission, and ubiquitination processes. The use of multiple algorithms enhances the comprehensiveness, robustness, and biological interpretability of the assessments, allowing for more accurate determination of mitophagy in ccRCC. Additionally, clusterProfiler (4.6.2) and fgsea (1.24.0) were applied for enrichment analysis of single-cell transcriptomic data of ccRCC, precisely assessing gene set enrichment for cell types such as clear cells, supporting queries to various biological databases including GO, KEGG, and Reactome (20, 21). CellChat R package (version 1.6.1) was utilized to analyze cell communication patterns (22). CellChat simulates cell communication based on interactions between signaling ligands, receptors, and auxiliary factors, revealing how cells collaborate. To compare metabolic state differences between normal and tumor tissues, this study used the scMetabolism package (version 0.2.1) for quantitative analysis of single-cell metabolic pathway activity. We also used the scFEA package to carry out flux studies to infer intracellular metabolites.

In this research, unsupervised non-negative matrix factorization (NMF) analysis of single-cell RNA sequencing data was applied using the NMF package (version 0.27) in R, aiming to explore the mitophagy characteristics of clear cell clusters (23). The component number was set to 10 to balance the granularity of different cell state distinctions and clustering interpretability. NMF results were integrated into the Seurat framework for dimensionality reduction clustering to identify different cell clusters. Key genetic markers were screened using the FindAllMarkers function, and each NMF cell cluster was categorized based on scores related to mitophagy-related genes and set thresholds. This method enhanced understanding of cell heterogeneity and tumor complexity, especially regarding mitophagy. Importantly, the ggplot2 package (version 3.4.2) served as our core tool for result visualization, offering a powerful and flexible way to create complex graphics based on the grammar of graphics.

### 2.3 Processing of spatial transcriptome sequencing data

In our study, the Seurat package (version 4.3.0) was used for the processing and analysis of spatial transcriptomics data, including normalization and feature selection of UMI counts with “SCTransform”, and dimensionality reduction with “RunPCA”. Additionally, the scMetabolism package was employed to assess metabolic features, while the “Monocle” package revealed cellular development and differentiation processes. In the Python environment, the Scanpy package processed spatial transcriptomics data through data preprocessing and dimensionality reduction with “SCTransform” and “RunPCA” (24). We also introduced the stLearn package, integrating gene expression, tissue morphology, and spatial location information to parse cell types, infer evolutionary paths, and identify cell interaction areas, providing a comprehensive spatial and functional perspective to understand tumor complexity (25).

### 2.4 Integrative analysis of spatial transcriptomics and single-cell sequencing data through deconvolution

Through deconvolution analysis, we inferred the proportions of cell types from mixed samples by combining single-cell and spatial transcriptomics data, revealing cellular and spatial heterogeneity within tissues. The “spacerxr” R package was used to perform RCTD analysis, constructing a reference model based on single-cell data and loading spatial data to form SpatialRNA objects. RCTD objects estimated the proportions of cell types in mixed samples through specific gene expression patterns, providing the distribution of cell types for each spot in the spatial data. Moreover, the “mistyR” package was employed to analyze cell interactions, revealing cellular interactions within tissues, inferring cell communication networks, and deepening the understanding of cell communication patterns in the tumor microenvironment (26).

### 2.5 Prognostic analysis of mitophagy-related clear cell subpopulations combined with bulk data

We explored the potential clinical prognostic value of newly identified mitophagy-related subpopulations of clear cells. For this purpose, we conducted an in-depth analysis using bulk sequencing data. Single-cell sequencing data were processed with the Seurat package, initially categorizing the identified mitophagy-related clear cells from patient tumor tissues into high and low expression subgroups based on their key gene expression levels. Next, the FindAllMarkers function was utilized to identify marker genes for these two subgroups. After obtaining the marker genes of key cell populations, we quantified these genes in bulk sequencing data, thus constructing high and low-risk groups. Lasso analysis was employed to filter out key prognostic genes for ccRCC, establishing a prognostic model based on mitophagy-related genes.

### 2.6 Cell culture and transient transfection

In our experimental studies, we utilized several cell lines, including the 786-O and 769-P renal clear cell carcinoma cells. These cell lines were obtained from the cell bank of the Central Laboratory at the Southwest Medical University Affiliated Hospital. To ensure the normal growth and maintenance of these cells, we cultured them in DMEM (HyClone) medium supplemented with 10% fetal bovine serum (HyClone), 100 U/L penicillin, and 100mg/L streptomycin (Thermo Fisher Scientific). We maintained standard culture conditions, including a 5% CO2 atmosphere, to provide an optimal environment for cell viability and experimental consistency. For the transient transfection experiments, we used Lipofectamine 3000 (Invitrogen, Carlsbad, CA, United States) as the transfection reagent. Negative control (NC) and UBB siRNA (RiboBio, Guangzhou, China) were transfected into the renal clear cell carcinoma cells according to the manufacturer’s instructions. This involved preparing a transfection mixture containing the siRNA and transfection reagent and then adding it to the cells. The transfection process was generally conducted within the recommended time frame according to the manufacturer’s protocol. By using Lipofectamine 3000 as the transfection reagent, our aim was to efficiently introduce the negative control or UBB siRNA into the renal clear cell carcinoma cells for subsequent analysis and research on the effects of gene knockdown or control on cellular processes and molecular pathways.

### 2.7 CCK-8 assay

We evaluated cell viability using the Cell Counting Kit-8 (CCK-8) assay. Twenty-four hours post-transfection, renal clear cell carcinoma cells were seeded into 96-well plates at a density of 1500 cells per well, and 200 μL of complete culture medium was added. The cells were then incubated at 37°C. For the CCK-8 assay, 10 μL of CCK-8 solution (Beyotime, Shanghai, China) was added to each well containing cells. After incubating for another 4 hours at 37°C, allowing the reagent to react with the cells, a colorimetric reaction related to cell viability occurs. At the end of the incubation period, the optical density (OD450) was measured using a microplate reader. The OD450 value reflects the absorbance of the formazan product generated by CCK-8, which is directly proportional to the metabolic activity and viability of the cells. By quantifying the OD450 values, we can assess the relative survival rate of the cells and compare them across different experimental conditions or treatment groups.

### 2.8 EdU-DAPI double staining assay

After 48 hours of transfection, 10 μM EdU was added and incubated for 4 hours, followed by fixation of cells with 4% paraformaldehyde for 10 minutes and permeabilization with 0.5% Triton X-100 for 5 minutes. EdU staining was performed using the Click-iT EdU Alexa Fluor 594 Imaging Kit according to the manufacturer’s instructions, followed by staining of cell nuclei with 1 μg/mL DAPI for 10 min. Finally, the cells were observed and images were acquired using fluorescence microscopy. Merge images were used to analyze cell proliferation.

### 2.9 Wound healing experiment

To evaluate the migration ability of renal clear cell carcinoma cells, we employed a wound healing assay. The transfected cells were cultured in six-well plates and maintained at 37°C until they reached approximately 80% confluence. A uniform wound was introduced into the cell monolayer using a 200 μl sterile pipette tip. After wound formation, the cells were washed twice with PBS to remove any debris, and then the medium was supplemented with serum-free culture medium. The process of cell migration into the damaged area was recorded at 0 hours and 24 hours using an Olympus inverted microscope.

### 2.10 Transwell assay

The invasive ability of renal clear cell carcinoma cells was assessed using a well-established technique in cell biology research—the Transwell assay. In this assay, a specific number of renal clear cell carcinoma cells (approximately 1 × 10^5) were seeded into specialized chambers. To evaluate invasion potential, chambers coated with Matrigel were used. The upper chamber contained serum-free culture medium to create a chemotactic gradient, while the lower chamber was filled with complete DMEM culture medium, providing a favorable environment for cell movement. After 24 hours of culture, cells that had successfully invaded through the membrane were fixed with a 4% paraformaldehyde solution. To observe and quantify the invaded cells, they were stained with 0.1% crystal violet. The stained cells were then observed and counted under an optical microscope, allowing for the assessment of cell numbers and invasion capability.

### 2.11 Statistical analysis

The statistical analyses were conducted using R version 4.2.2, 64-bit, along with its support packages. The pycharm integrated development environment for Python was also utilized. The non-parametric Wilcoxon rank sum test was employed to assess the relationship between two groups for continuous variables. Spearman correlation analysis was conducted to examine correlation coefficients. A significance level of P<0.05 was considered statistically significant for all statistical investigations.

## 3 Results

### 3.1 Single-cell transcriptome atlas of clear cell renal cell carcinoma

**Figure 1:**
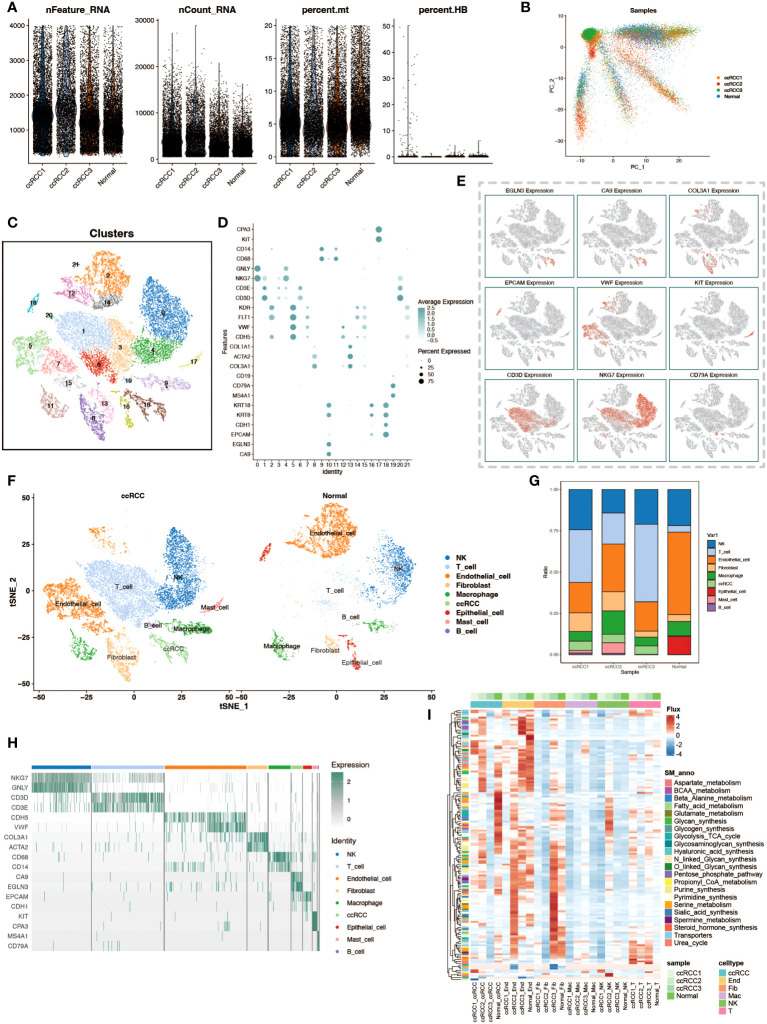
Single-cell transcriptomic atlas analysis of renal clear cell carcinoma. **(A)** Data quality control. Violin plots depict the number of genes per cell (nFeature_RNA), total transcript counts (nCount_RNA), percentage of mitochondrial genes (percent.mt), and percentage of hemoglobin genes (percent.HB) to evaluate sample quality. **(B)** PCA dimensionality reduction of patient samples. Principal component analysis (PCA) results based on expression profiles show the distribution of cell populations from different patients (ccRCC for tumor tissues of renal clear cell carcinoma patients, Normal for normal adjacent tissue samples). **(C)** t-SNE clustering visualization. The t-SNE dimensionality reduction technique reveals 22 distinct cell populations, each identified by a different color. **(D)** Marker genes of cell populations. Bubble charts display selected marker genes expressed in different cell populations. **(E)** Spatial expression patterns of cell marker genes. The t-SNE plot shows the expression patterns of selected marker genes. **(F)** Comparison of cell types between tumor and normal groups. The t-SNE plot shows the distribution of cell populations from the tumor and normal groups. **(G)** Stacked bar charts display the proportion of cell type distribution across different patient samples. **(H)** Heatmap of marker gene expression. Displays the expression levels of specific marker genes in different cell types. **(I)** Heatmap of metabolic levels. Shows how active various cell types are in different metabolic pathways in different samples.

In this study, we delved into the cellular heterogeneity and composition of ccRCC and its adjacent normal kidney tissue through scRNA-seq. To ensure the quality of data and rigor of analysis, we first performed meticulous quality control, quantifying multiple quality metrics including the assessment of the number of feature genes per cell, UMI counts, and the percentage of mitochondrial and hemoglobin gene expression, thereby eliminating the interference of senescent cells and erythrocytes (**Figure 1A**). Subsequently, we utilized the Harmony package for batch effect correction based on PCA analysis, which ensured the reliability of the analysis results while maximally preserving the original gene expression information of the cells (**Figure 1B**). By using the t-SNE algorithm, we performed a visualization of the cell clustering results, showing 22 cell clusters (**Figure 1C**). Based on the cell marker genes, we plotted bubble plots and feature plots to help us identify cell types by the expression and expression distribution of these genes (**Figures 1D, E**). After completing the cell type identification, we compared the cell distribution and number in ccRCC samples and normal kidney tissue samples, and observed that there was a significant increase in the proportion of T cells in ccRCC tissues (**Figures 1F, G**). The expression of cell marker genes in various cell types of cells is demonstrated by gene expression heatmap to check the accuracy of cell type identification (**Figure 1H**). To gain a preliminary understanding of the metabolic functionality of various cell types in tumor and normal tissues, we performed flux estimation analysis to infer intracellular fluxes of metabolites (**Figure 1I**).

### 3.2 Exploring mitophagy levels in ccRCC by gene scoring

**Figure 2:**
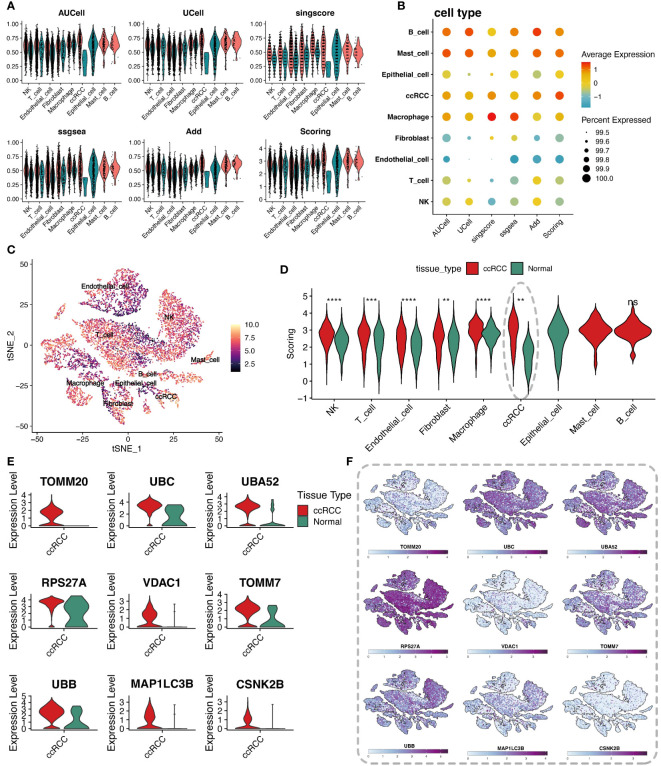
Analysis of cellular metabolic levels. **(A)** Violin plots of mitochondrial autophagy gene set scores through five gene set scoring methods and an integrated score. **(B)** Bubble chart of mitochondrial autophagy gene set expression scores in different cell types, based on the expression level of specific gene sets. **(C)** t-SNE plot showing the distribution of metabolic scores among cells, where the depth of color represents the level of scoring, revealing the metabolic heterogeneity of different cell types. **(D)** Violin plots comparing mitochondrial autophagy scores differences between tumor tissues and adjacent normal tissues for each cell type, showing changes in mitochondrial autophagy states in the tumor microenvironment. **(E)** Violin plots of differentially expressed mitochondrial autophagy genes between tumor and normal groups. **(F)** t-SNE plot showing the heatmap of differentially expressed mitochondrial autophagy genes expression levels in different cell types. "*" represents p-value less than 0.05, "**" represents p-value less than 0.01, "***" represents p-value less than 0.001. "****" represents p-value less than 0.0001. "ns" represents not statistically significant (p ≥ 0.05).

To delve into the regulatory mechanisms of mitophagy in ccRCC and its role in the pathological process, this study quantitatively evaluated the activity of mitophagy genes in ccRCC from multiple perspectives using various scoring algorithms, including AUCell, UCell, singcore, ssgsea, and AddModuleScore, revealing their potential role in tumor development. The analysis vividly presented the scores of mitophagy gene sets across various cell types through violin plots and bubble charts (**Figures 2A, B**). Heatmaps displayed the final scores for different cell types (**Figure 2C**). Comparing the scores of cells from different groups and performing Wilcox statistical analysis revealed that the scores of clear cells in the tumor group were significantly higher than those in the normal group, with statistically significant differences (p-value < 0.05) (**Figure 2D**). To further reveal which mitophagy genes play a key role in the pathogenesis of ccRCC, differential analysis was conducted between the tumor group and its normal counterpart, intersecting the resultant differential genes with mitophagy-related genes, and obtaining 9 key mitophagy-related genes with a logFC threshold of 0.5. The results showed that TOMM20, UBC, UBA52, RPS27A, and other genes were significantly upregulated in ccRCC cells (**Figure 2E**). Notably, these genes were not only universally upregulated in ccRCC cells but also widely distributed across various cell subpopulations (**Figure 2F**).

### 3.3 Characteristics of renal clear cells in the high and low mitophagy level group

**Figure 3:**
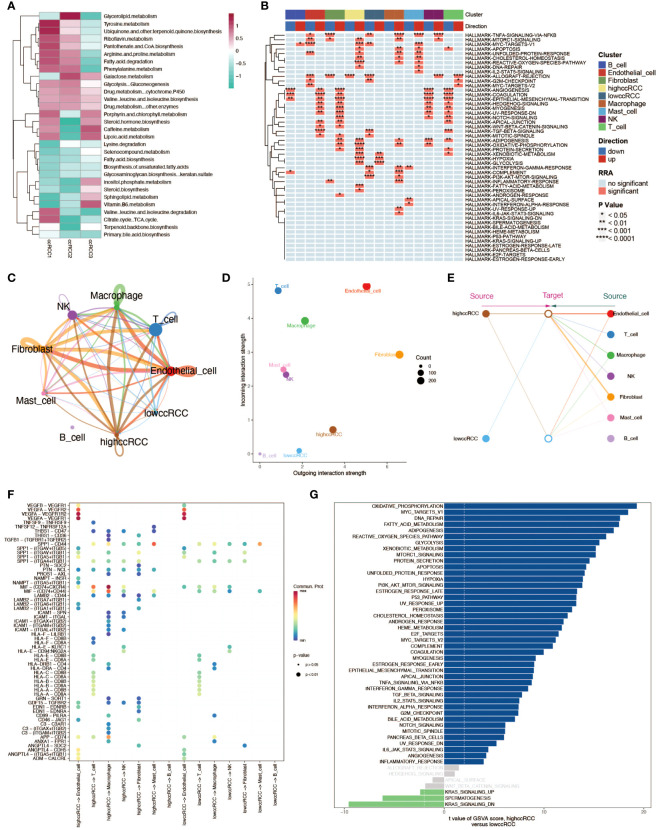
Multidimensional analysis of renal clear cells. **(A)** Heatmap of metabolic activity in renal clear cells from different sample sources, analyzed using the scMetabolism package, showing their expression patterns across different metabolic pathways. **(B)** HALLMARK gene set enrichment analysis. Individual cell types (including high and low group ccRCC) are shown to be up- and down-regulated in various pathways. **(C)** String diagram of cellular communication networks. Demonstrates the strength of cellular communication in tumor tissues. **(D)** Scatter plot demonstrating the average strength of signals received and sent by cells in each cell type. **(E)** Structural diagram demonstrating the communication patterns of various cell types in the cellular communication network, comparing the high and low groups of ccRCC. **(F)** Ligand receptor activation involved in cellular communication between high and low groups of ccRCC and other cell types. **(G)** GSVA enrichment analysis of evanescent bar graphs. Pathways with significant differences between the high-level group ccRCC and the low-level group are demonstrated.

We first analyzed the metabolic pathway activity in three ccRCC samples to determine the metabolic characteristics of ccRCC (**Figure 3A**). In these three tumor sample data, we divided renal clear cells into high and low groups according to the median value of his mitochondrial autophagy score to explore the effect of mitophagy levels on renal clear cell function and activity. Enrichment analyses showed a very significant difference in the functional activity of renal hyalinocytes between the high and low groups (**Figure 3B**). There were also differences in cellular communication between the high and low groups of renal hyalocytes, with the high mitophagy level group having a higher level of cellular communication than the low level group, both in terms of signaling efference and signaling reception, as well as differences in the structure of the communication patterns between the two groups (**Figures 3C–E**). The signaling pathways ligand receptors they involve also differ markedly in type and strength (**Figure 3F**). Differences between the two groups of cells were more clearly demonstrated by GSVA enrichment analyses, with renal hyalinocytes generally functioning more actively in the high-level group than in the low-level group (**Figure 3G**).

### 3.4 Application of non-negative matrix factorization (NMF) in revealing heterogeneity of mitophagy in renal clear cells

**Figure 4:**
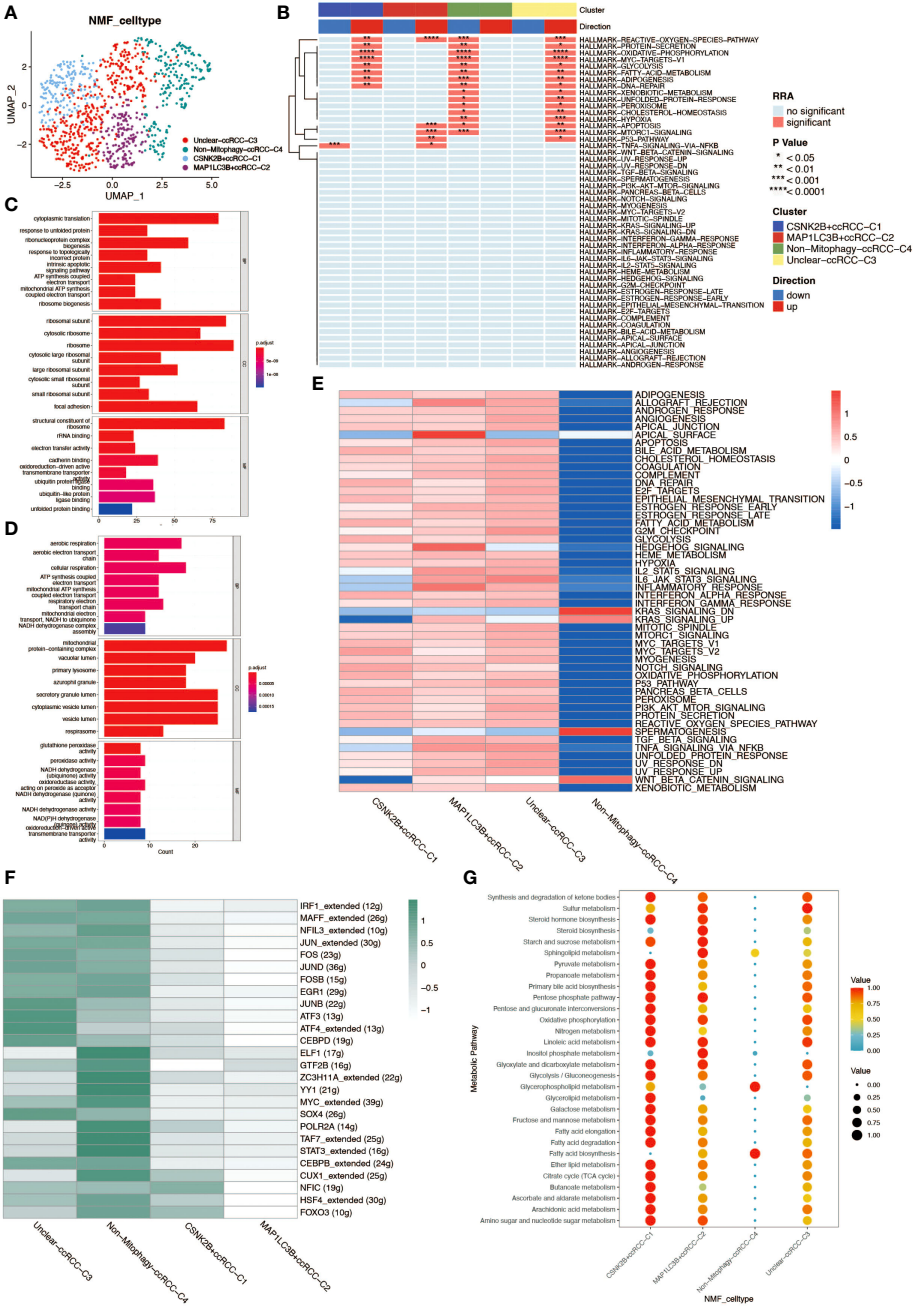
Non-negative matrix factorization (NMF) analysis of renal clear cells. **(A)** UMAP plot annotating renal clear cell types post-NMF, identifying two subtypes closely related to mitochondrial autophagy. **(B)** HALLMARK gene set enrichment analysis. The up- and down-regulation of the four ccRCC subgroups obtained from NMF analysis in various pathways is shown. **(C)** GO enrichment analysis of cell population C1. results of GO enrichment analysis of CSNK2B+ccRCC-C1 cell population demonstrating properties in BP, CC, and MF. **(D)** GO enrichment analysis of cell population C2. results of GO enrichment analysis of MAP1LC3B+ccRCC-C2 cell population, demonstrating the properties in three aspects: BP, CC, and MF. **(E)** GSVA enrichment analysis. The heatmap demonstrates the differences between the four ccRCC subpopulations in various pathways. **(F)** Heatmap of the transcription factor regulatory network in mitochondrial autophagy-related subtypes of renal clear cells. **(G)** Bubble chart analyzing the activity levels of renal clear cell subgroups in different metabolic pathways, where bubble size and color depth reflect the relative levels of metabolic activity.

Non-negative matrix factorization (NMF) is a matrix decomposition method performed under the constraint that all elements of the output matrices are non-negative. Compared to principal component analysis (PCA), NMF has a natural advantage in analyzing tumor cell heterogeneity. By applying NMF technology and clustering ccRCC cells based on mitochondrial autophagy-related genes, we successfully identified five distinct subgroups (C0–4). To elucidate the potential link between the subgroups obtained by NMF analysis and mitochondrial autophagy, we performed differential expression analyses of cells in these subgroups. We obtained the differentially expressed genes for each subgroup and developed a series of rules to identify cell types: 1.differentially expressed genes were ranked according to logFC values. 2.If the first gene was a mitochondrial autophagy-related gene with a logFC value greater than 1 and a P value of less than 0.05, then the cell population was defined as a cell population marked by this gene. 3. If the first gene is a mitochondrial autophagy-related gene but its logFC value is less than 1 or its P value is greater than 0.05, then the cell population cannot be defined. 4. if the first gene is not a mitochondrial autophagy-related gene, then the cell population is defined as a non-mitochondrial autophagy cell population (Non-Mitophagy). With this approach, we were able to identify and categorize cell populations more clearly. The results yielded four subgroups: Unclear-ccRCC-C3, Non-Mitophagy-ccRCC-C4, CSNK2B+ccRCC-C1, MAP1LC3B+ccRCC-C2 (**Figure 4A**). Among them, Unclear-ccRCC-C3 was named due to the most significant gene logFC not meeting the selection criteria, and the Non-Mitophagy-ccRCC-C4 subgroup’s most significant gene was not a mitochondrial autophagy gene. We performed a series of analyses on the four cell subpopulations of renal clear cells obtained to investigate differences in functional activity and biological heterogeneity. The results of the Hallmark enrichment analysis showed that the Non-Mitophagy cell population (C4) was significantly different from the remaining three populations, which happen to be the ones that are or may be related to mitophagy (**Figure 4B**). GSVA enrichment analysis also demonstrated concordant results, which further demonstrated the accuracy of the NMF analysis in identifying the mitophagy renal hyalinocyte subpopulation (**Figure 4E**). For the two identified populations of mitophagy -associated renal clear cells (CSNK2B+ccRCC-C1 and MAP1LC3B+ccRCC-C2), we performed separate GO enrichment analyses for further exploration of these two key cell types (**Figures 4C, D**). In the transcription factor analysis, opposite results were presented, with a stronger relationship between the Non-Mitophagy cell population and the transcription factors (**Figure 4F**). The cellular metabolic profiles in the four cell subpopulations demonstrated very clear differences between Non-Mitophagy and mitophagy -associated renal hyalinocytes, with mitophagy -associated renal hyalinocytes being much more advanced than the Non-Mitophagy cell population in a variety of metabolic pathways (**Figure 4G**).

### 3.5 Analysis of metabolic features in spatial transcriptomics data

**Figure 5:**
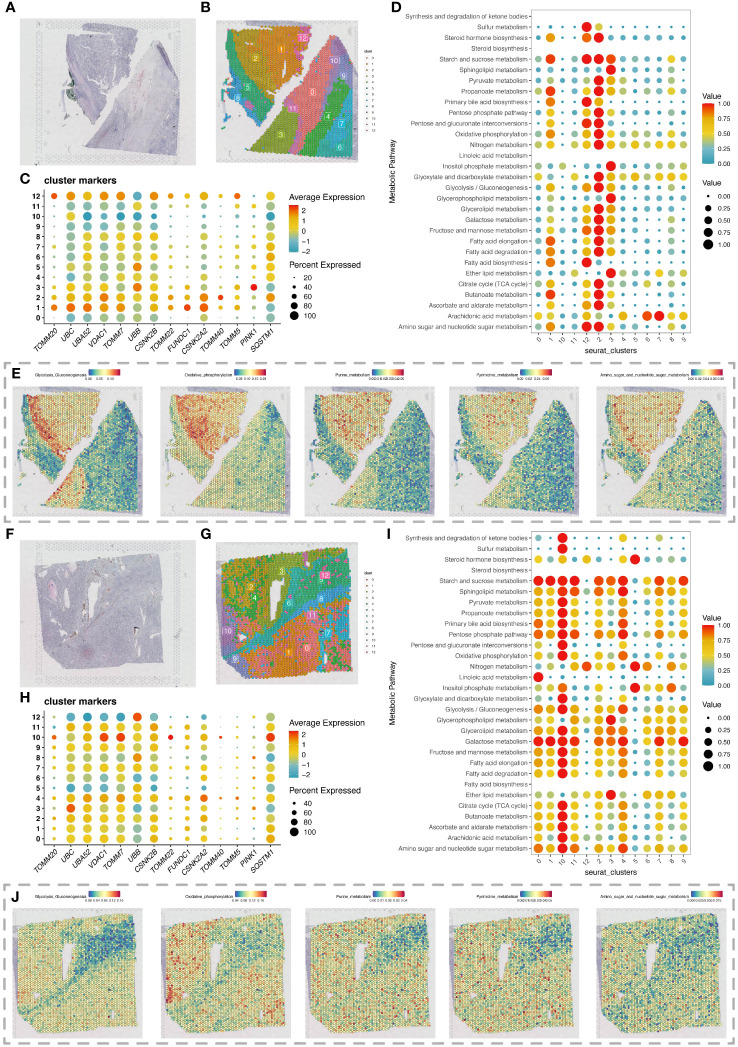
Spatial transcriptomics analysis revealing changes in metabolic activity. **(A)** H&E-stained section of renal clear cell carcinoma tumor tissue. **(B)** Spatial transcriptomics data of a renal clear cell carcinoma tumor tissue section, with cell clustering results obtained by dimensionality reduction clustering analysis using the Seurat package. **(C)** Expression of differentially expressed mitochondrial autophagy-related genes in the section data. **(D)** Bubble chart showing metabolic activity levels of different cell clusters in the renal clear cell carcinoma tumor tissue section analyzed with the scMetabolism package, highlighting each cluster’s performance in various metabolic pathways. **(E)** Display of various metabolic levels on the section, including glycolysis, oxidative phosphorylation, purine metabolism, pyrimidine metabolism, and the metabolism of amino sugar and nucleotide sugar. **(F)** Second H&E-stained section of renal clear cell carcinoma tumor tissue. **(G)** Spatial transcriptomics data of a second renal clear cell carcinoma tumor tissue section, with cell clustering results obtained by dimensionality reduction clustering analysis using the Seurat package. **(H)** Expression of differentially expressed mitochondrial autophagy-related genes in the second section data. **(I)** Bubble chart showing metabolic activity levels of different cell clusters in the second renal clear cell carcinoma tumor tissue section analyzed with the scMetabolism package, highlighting each cluster’s performance in various metabolic pathways. **(J)** Display of various metabolic levels on the second section, including glycolysis, oxidative phosphorylation, purine metabolism, pyrimidine metabolism, and the metabolism of amino sugar and nucleotide sugar.

Spatial transcriptomics data provided HE stained slice images of two ccRCC tumor tissue samples (**Figures 5A, F**). After dimensionality reduction clustering of spatial transcriptomics data, we mapped the clustering information onto the HE stained slices, obtaining dimensionality reduction clustering maps on the slices (**Figures 5B, G**). The differential expression of mitochondrial autophagy genes between tumor and normal groups in single-cell data was displayed on spatial transcriptomics data through bubble charts (**Figures 5C, H**). By using the scMetabolism package for metabolic analysis of spatial transcriptomics data, we showed the specific metabolic levels of each cell cluster in the two tumor samples (**Figures 5D, I**) and also mapped certain key metabolisms onto the slices. The heatmap colors allowed us to clearly see the high and low states of metabolism at different locations on the slices (**Figures 5E, J**).

### 3.6 Pseudotime analysis of spatial transcriptomics

**Figure 6:**
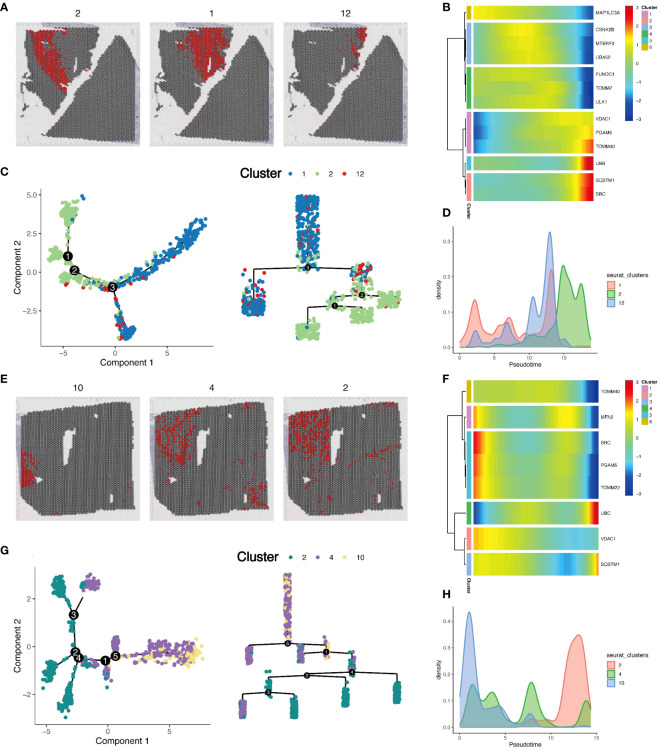
Pseudotime analysis of cells in local areas of tumor tissue sections. **(A)** Display of local cell populations on the section. **(B)** Expression of mitochondrial autophagy-related genes in pseudotime order. **(C)** Developmental trajectory map, showing the dynamic changes and differentiation paths of 3 cell populations in pseudotime development. **(D)** Density map explaining the distribution characteristics of each group of cells on the pseudotime axis. **(E)** Display of local cell populations on the section in a second renal clear cell carcinoma tumor slice. **(F)** Expression of mitochondrial autophagy-related genes in pseudotime order on the second slice. **(G)** Developmental trajectory map for the second slice data, showing the dynamic changes and differentiation paths of 3 cell populations in pseudotime development. **(H)** Density map explaining the distribution characteristics of each group of cells on the pseudotime axis for the second slice.

In **Figures 5E, J**, we observed high metabolic areas on the slices of two tumor samples, with clusters 2 and 1 being the main high metabolic areas on the first slice, and clusters 10 and 4 on the second slice. Therefore, we selected the high metabolic areas and their surrounding cells for pseudotime analysis using the Monocle package. For the first slice, we conducted pseudotime analysis on cell clusters 2, 1, and 12 (**Figure 6A**). The heatmap showed the expression changes of mitochondrial autophagy-related genes over pseudotime (**Figure 6B**). Cluster 1 occupied the earliest branch in the pseudotime analysis, while cluster 2 was on a later branch, which might indicate the developmental sequence of tumor cells (**Figure 6C**). The cell density map also hinted at the timing of cell appearances (**Figure 6D**). For the second slice, we analyzed cell clusters 10, 4, and 2, with the heatmap showing the expression changes of mitochondrial autophagy-related genes over pseudotime (**Figures 6E, F**). Cell clusters 10 and 4 were primarily in the early stages of the pseudotime sequence, while cluster 2 was mainly in the later stages (**Figures 6G, H**).

### 3.7 Developmental trajectories revealed by spatial transcriptomics data

**Figure 7:**
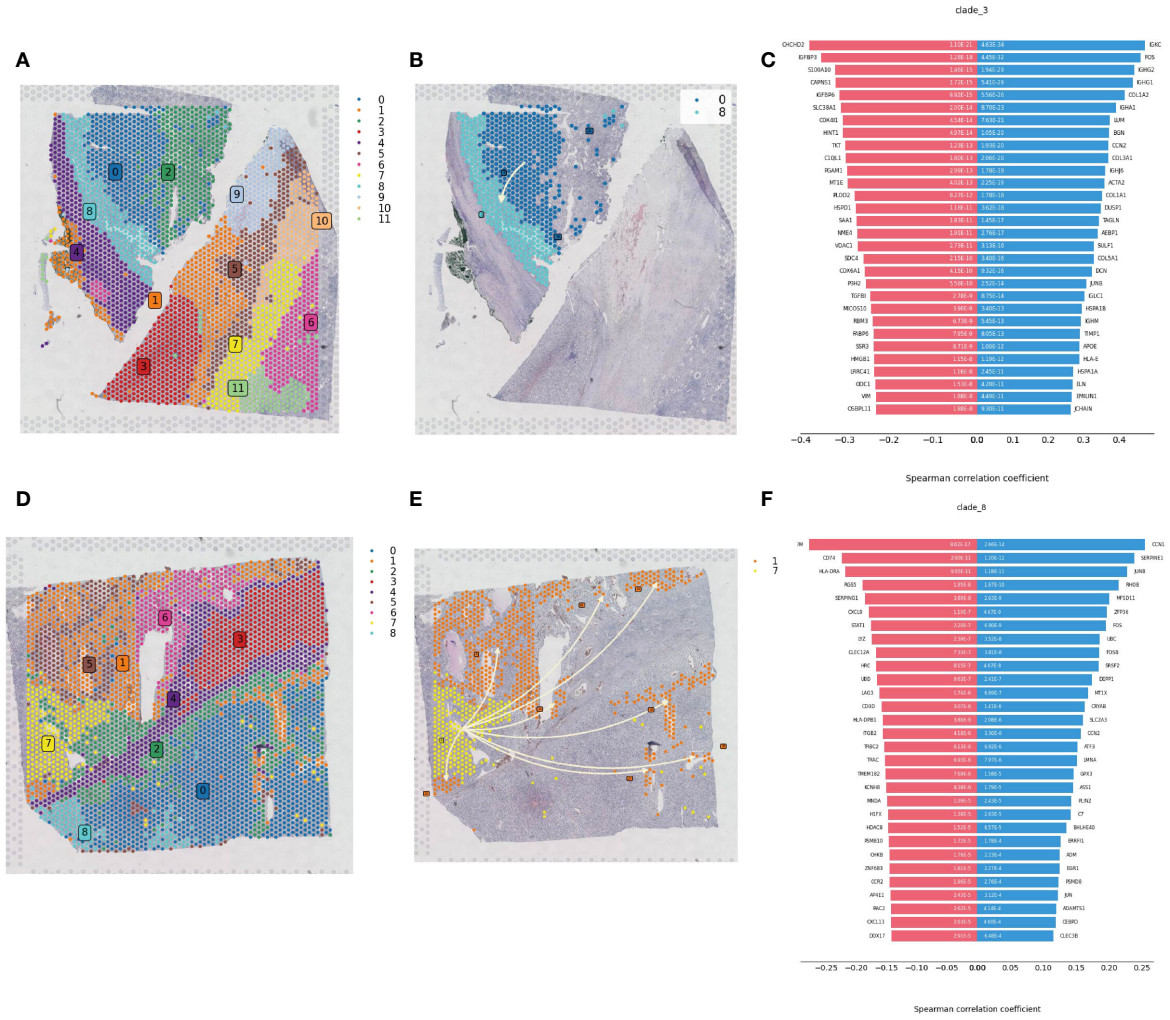
Spatial developmental trajectory analysis of renal clear cell carcinoma tumor tissue. **(A)** Clustering of renal clear cell carcinoma tumor tissue section sequencing data using the louvain method in the stLearn package, with the clustering map showing the spatial distribution of different cell populations. **(B)** Spatial developmental trajectory map of high metabolic area cells in the tumor tissue section, drawn using the stLearn package. **(C)** Diverging bar chart of developmental trajectory-related differentially expressed genes in the tumor tissue, performed statistical analysis using numpy, revealing key regulatory genes associated with the developmental trajectory. **(D)** Clustering map of the second slice data. **(E)** Spatial developmental trajectory map of high metabolic area cells. **(F)** Diverging bar chart of developmental trajectory-related differentially expressed genes in the tumor tissue.

Spatial transcriptomics data provide transcriptional information on the precise location of cells within tissues. Using the stLearn toolkit, we conducted an in-depth analysis of spatial transcriptomics data to explore the developmental processes of tumors, including invasion and metastasis issues. By combining data quality control and dimensionality reduction with NumPy, and clustering with stLearn’s Louvain method, we identified different cell clusters in ccRCC samples (**Figures 7A, D**). For cell clusters identified in the early stages of pseudotime sequence in the pseudotime analysis, we reconstructed the developmental trajectories using the Diffusion Pseudotime (DPT) algorithm, combined with spatial coordinates information, revealing the gradual invasion and metastasis process of tumor cells in the pseudotime sequence, consistent with Monocle pseudotime analysis (**Figures 7B, E**). The diverging bar charts of developmental trajectory analysis revealed gene expression changes based on trajectory differences, showing genes that were upregulated and downregulated throughout the tumor development process from start to end (**Figures 7C, F**).

### 3.8 Deconvolution and cell interaction analysis combining spatial transcriptomics with single-cell data

**Figure 8:**
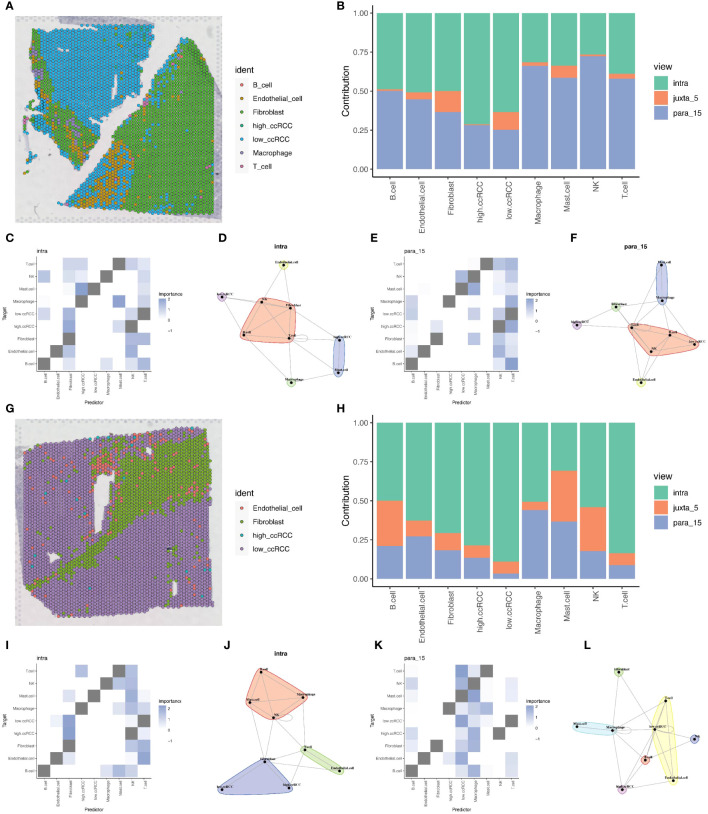
Deconvolution and cell interaction analysis based on spatial transcriptomics data. **(A)** Analysis of renal clear cell carcinoma tumor tissue section data using the RCTD deconvolution method, showing the spatial distribution probabilities of various cell types, including cells with high and low levels of mitochondrial autophagy. **(B)** Bar chart showing the contribution of different views to cell interactions assessed by the Mistyr package, demonstrating the relative importance of different views in cell interactions. **(C, D)** Heatmap and network diagram of cell interactions within the same view (intraview), revealing the interaction strength and patterns within the same cell type. **(E, F)** Heatmap and network diagram of cell interactions in the paraview15 view, showing the interaction strength and communication networks across cell types. **(G)** RCTD deconvolution analysis results of the second slice data, showing the probabilities and spatial distribution of different cell types, including cells with high and low levels of mitochondrial autophagy. **(H)** Bar chart showing the contribution of different views to cell interactions in prostate adenocarcinoma with infiltrating carcinoma tissue, assessing the relative contributions of each view. **(I, J)** Heatmap and network diagram of cell interactions (intraview) for the second slice data, showing the interaction relationships among the same cell type in the tumor environment. **(K, L)** Heatmap and network diagram of cell interactions in the paraview15 view of the same tissue, revealing the interaction strength and network structures across different cell types.

Due to the limitations of spatial transcriptomics sequencing technology, current spatial transcriptomics data do not achieve the same single-cell resolution as single-cell sequencing data. To address the limitations of spatial transcriptomics sequencing data, we employed deconvolution analysis methods to compensate for its lack of resolution. This analysis inferred the possible cell types and their proportions at each location in the spatial transcriptomics data based on the gene expression patterns of various cell types in ccRCC single cell sequencing data. This step allowed us to gain deeper insights into the spatial structure and function of tissues or cells, revealing interactions and communications between different cell types, and discovering spatial heterogeneity and state changes of cells. Through this method, we were able to provide more detailed information about cell types and proportions in ccRCC tumor samples, offering new perspectives and depth to the study (**Figures 8A, G**). Based on the deconvolution analysis of two tumor samples, we further applied the MISTy (Multiview Intercellular SpaTial modeling framework) framework for spatial transcriptomics cell interaction analysis. This framework is an interpretable machine learning framework for analyzing single-cell, highly multiplexed, spatially resolved data, enabling an in-depth understanding of the internal and intercellular relationships between markers. With MISTy, we could handle a custom number of views, each describing different spatial contexts such as intracellular regulation or paracrine regulation, and relationships between specific cell types. Our analysis results showed the contributions of three different views to cell interactions through bar charts, finding that intraview and paraview15 made the largest contributions in the two tumor samples (**Figures 8B, H**). This revealed the importance of intracellular regulation and paracrine regulation in tumor samples. Further heatmap and network graph analyses revealed the specific patterns of these two views in tumor samples, highlighting the significant interactions between two groups of clear cells with high and low mitochondrial autophagy states and other cell types (such as mast cells and fibroblasts) (**Figures 8C–F, I–L**).

### 3.9 Prognostic study of mitochondrial autophagy-related genes

**Figure 9:**
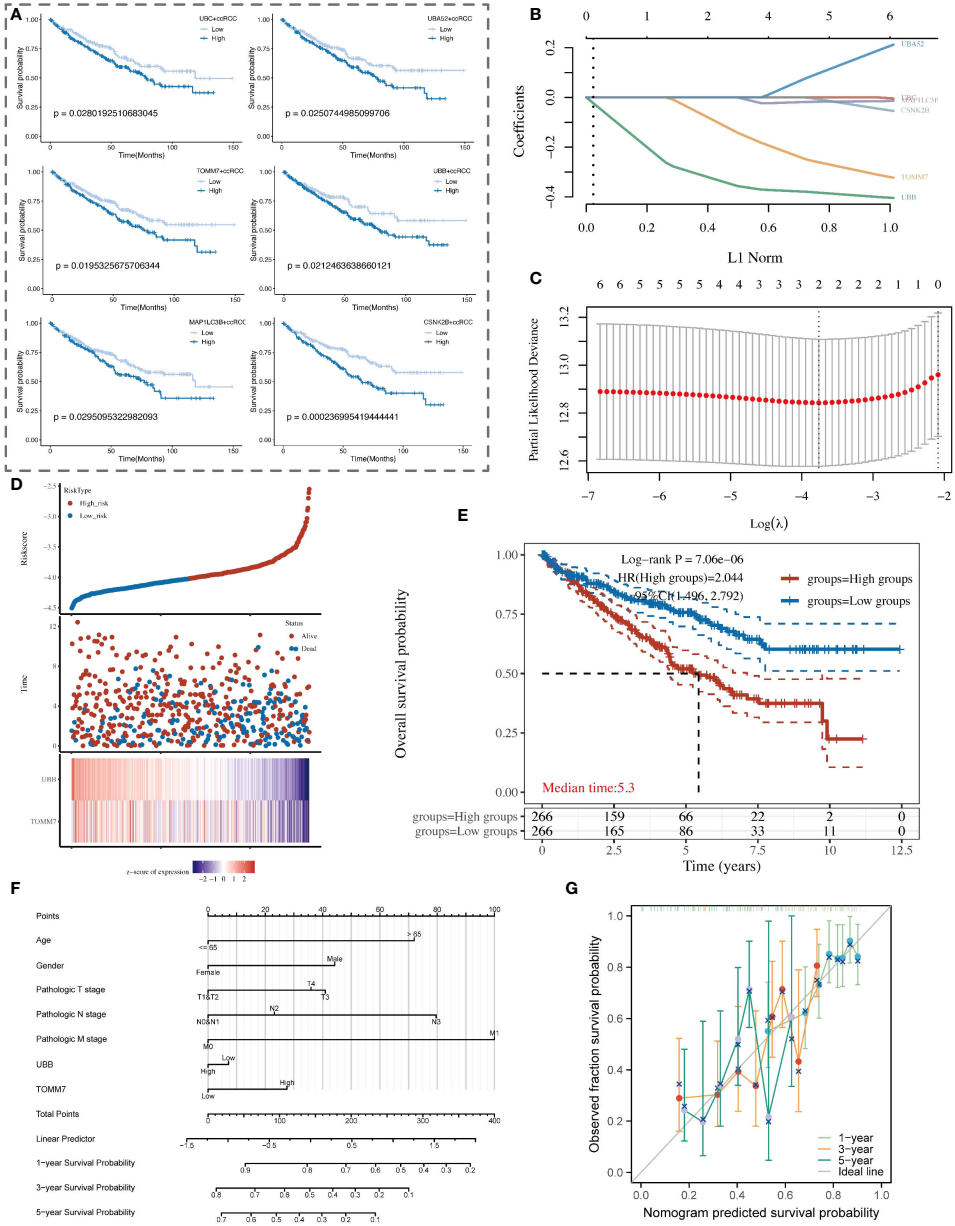
Analysis of the association between mitochondrial autophagy-related subtypes of renal clear cells and the clinical prognosis of patients with renal clear cell carcinoma. **(A)** Kaplan-Meier survival curve analysis showing the survival probability differences among renal clear cell subtypes with high and low expression of key mitochondrial autophagy genes in patients with renal clear cell carcinoma. **(B)** LASSO coefficient path graph. It illustrates how the LASSO coefficients of renal clear cell carcinoma prognosis-related genes change as the regularization strength (L1 norm) of the model increases. Selected genes maintain non-zero coefficients at high regularization levels, indicating their importance to the model. **(C)** Deviance plot of ten-fold cross-validation. Displays the performance of the LASSO model at different lambda values to determine the optimal lambda selection. The red dot identifies the lambda value providing the optimal prognosis model, determined by minimizing the validation error. **(D)** Risk score and survival status chart of patients with renal clear cell carcinoma based on the LASSO model. **(E)** Kaplan-Meier survival analysis curve of high and low-risk patient groups with renal clear cell carcinoma. **(F)** Nomogram model for the prognosis of patients with renal clear cell carcinoma, combining clinical variables such as age, gender, pathological staging, and the expression levels of UBB and TOMM7 genes. **(G)** Calibration curve of the nomogram prognosis model, showing the consistency between the nomogram-predicted 1-year, 3-year, and 5-year survival probabilities (X-axis) and the actual observed survival probabilities (Y-axis).

In our study, nine key mitochondrial autophagy-related genes were significantly higher expressed in tumor tissues compared to normal tissues. We analyzed data of ccRCC from the TCGA database, first selecting positive cells with high expression of these nine genes, and compared them with negative cells with low expression to identify unique marker genes of the positive cells. Subsequently, based on the expression levels of these marker genes, we divided patients into high and low expression groups and performed survival analysis. The results showed that patients with high expression of UBC, UBA52, TOMM7, UBB, MAP1LC3B, and CSNK2B had a poorer prognosis, with statistical significance (**Figure 9A**). Using LASSO regression model analysis, UBB and TOMM7 were identified as important prognostic factors for ccRCC (**Figures 9B–D**). Kaplan-Meier curves showed that the survival rate of patients in the high-expression group was significantly lower than that of the low-expression group (**Figure 9E**). We also established a nomogram that includes these genes and clinical parameters to predict the survival probabilities of patients at 1, 3, and 5 years, and calibration curves validated the accuracy and reliability of this prognostic model (**Figures 9F, G**).

### 3.10 UBB promotes the proliferation and migration of renal clear cell carcinoma cells

**Figure 10:**
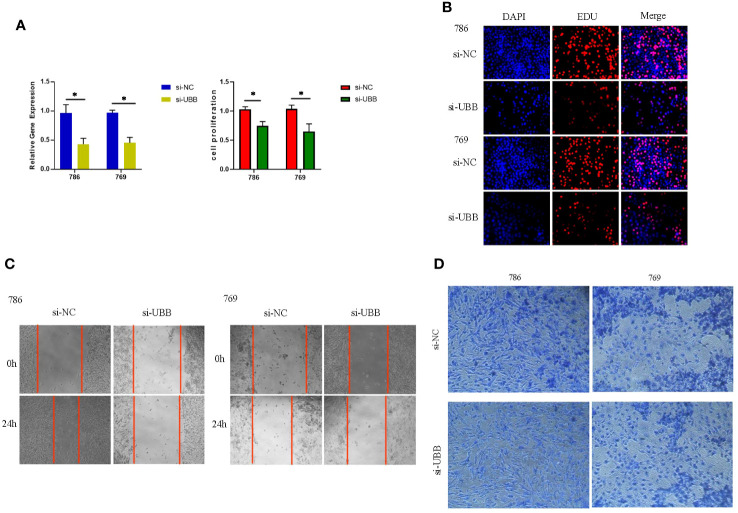
UBB has been demonstrated to promote the proliferation, invasion, and migration of renal clear cell carcinoma cells, as determined by a series of analytical results: **(A)** CCK-8 assay. **(B)** EdU-DAPI Double Staining Assay. **(C)** wound healing assay. **(D)** Transwell assay. * Indicates p-value < 0.05.

To investigate the potential role of UBB in renal clear cell carcinoma, we conducted *in vitro* experiments. Initially, the CCK-8 assay indicated that silencing UBB significantly inhibited cell proliferation (**Figure 10A**). Silencing of the UBB gene resulted in a significant reduction in DAPI staining (blue) and EdU staining (red) signals in the 786 and 769 cell lines, indicating a decrease in both the number of cells and the number of DNA-synthesizing cells. The results showed that UBB gene knockdown significantly inhibited the proliferation of tumor cells (**Figure 10B**). Furthermore, wound healing assays, and transwell assays showed that knocking out UBB significantly reduced the cells’ invasion and migration capabilities (**Figures 10C, D**). Taken together, these results suggest that the upregulation of UBB promotes the proliferation, invasion, and migration of renal clear cell carcinoma cells.

## 4 Discussion

Mitochondrial defects, including structural or functional abnormalities caused by genetic mutations, damage from environmental factors, increased oxidative stress, or mitochondrial DNA (mtDNA) damage, impact cellular proliferation, death, and metabolism and are closely linked to the development and progression of cancer (27, 28). These defects can trigger mitophagy—a cellular adaptive mechanism that maintains cell survival by removing dysfunctional mitochondria to prevent further cellular damage. As a quality control mechanism, mitophagy aids in the clearance of unhealthy or dysfunctional mitochondria, averting potential cellular damage caused by mitochondrial defects (12, 29). With an increase in mitochondrial defects, autophagy activity correspondingly intensifies to address these deficiencies. The process of mitophagy includes multiple steps: recognition of mitochondrial damage, formation of autophagosomes, fusion with lysosomes, degradation, and recycling (12). In our research, we observed changes in the levels of mitochondrial autophagy in various cell types within ccRCC tumor tissues compared to normal kidney tissue, especially a significant enhancement of mitochondrial autophagy levels in clear cells within tumor groups.

The enhancement of mitochondrial autophagy in ccRCC tissues can be understood from multiple perspectives: Firstly, tumor cells undergo metabolic reprogramming to adapt to the tumor microenvironment and promote survival and proliferation, activating more frequent mitochondrial autophagy to maintain intracellular metabolic balance. Secondly, ccRCC cells may experience increased oxidative stress, leading to mitochondrial damage, and enhance mitochondrial autophagy to clear damaged mitochondria, preventing the accumulation of oxidative damage that could lead to cell death. Additionally, mitochondrial autophagy may serve as a self-regulatory mechanism, helping tumor cells optimize survival strategies to adapt to stress conditions in the microenvironment (30, 31).

Through the analysis of multiple transcriptomic data, we identified several key genes closely related to mitochondrial autophagy, suggesting that these genes may be the main factors driving the changes in mitochondrial autophagy function in clear cells of renal cell carcinoma, especially in the prognostic analyses the high expression of six mitochondrial autophagy-related genes, namely, UBC, UBA52, TOMM7, UBB, MAP1LC3B, and CSNK2B was closely associated with poor patient prognosis. Among these genes, UBC (Ubiquitin C), UBA52 (Ubiquitin A-52 Residue Ribosomal Protein Fusion Product 1), and UBB (Ubiquitin B) are involved in the ubiquitination process—a critical protein modification mechanism that tags proteins for degradation or other fates (32). The ubiquitin-proteasome system plays a central role in regulating protein levels, maintaining protein homeostasis, and participating in cellular stress responses. TOMM7 (Translocase Of Outer Mitochondrial Membrane 7) is part of the mitochondrial protein import complex, responsible for transporting proteins from the cytosol into the mitochondria (33). MAP1LC3B (Microtubule Associated Protein 1 Light Chain 3 Beta) is a key protein in the autophagy process, involved in the formation of autophagosomes (34). CSNK2B (Casein Kinase 2 Beta), as part of the protein kinase CK2, is involved in various cellular processes including cell cycle regulation, cell survival, and DNA repair (35). Further analyses identified UBB and TOMM7 as important prognostic factors for ccRCC.

UBB is a protein-coding gene involved in the process of ubiquitination and is also associated with mitochondrial autophagy. The ubiquitination process plays a critical and widespread regulatory role within the cell, maintaining the stability of the intracellular environment and responding to environmental changes by controlling the fate of proteins. In mitochondrial autophagy, ubiquitination plays a central role, primarily by covalently attaching ubiquitin proteins to specific proteins on the surface of damaged or dysfunctional mitochondria, thereby marking these mitochondria for recognition and clearance by autophagosomes. The involvement of specific receptor proteins such as p62/SQSTM1, OPTN, and NBR1 allows these ubiquitinated mitochondria to interact with LC3 proteins on the autophagosome membrane, promoting the formation and expansion of autophagosomes to encapsulate and ultimately digest the damaged mitochondria (12, 29). Specifically, the ubiquitin B protein encoded by the UBB gene plays a core role in marking damaged or obsolete proteins for recognition and degradation by the 26S proteasome. By regulating the selective degradation of mitochondria, the UBB gene and its encoded ubiquitin B protein are crucial for maintaining mitochondrial quality control and intracellular environmental stability. High expression of the UBB gene may enhance the ubiquitination marking and rapid clearance of damaged mitochondria, helping tumor cells effectively remove damaged mitochondria to prevent cellular stress and death, thereby increasing the tumor cells’ adaptability to adverse conditions. Furthermore, high expression of the UBB gene may also strengthen the adaptive response of the autophagy pathway under stress conditions such as nutrient deprivation or hypoxia, providing a survival advantage for tumor cells, especially in the challenging tumor microenvironment.

We have demonstrated through *in vitro* experiments that the proliferation, invasion and migration of tumor cells can be inhibited by decreasing the expression of the UBB gene in tumor cells. The results based on transcriptome data analysis and *in vitro* experiments demonstrated that UBB, a mitochondrial autophagy-related gene, has a very important role in renal clear cell carcinoma, which provides a new direction for potential clinical treatment. We can envisage the development of siRNA drugs or small molecule inhibitors based on the UBB gene, thereby reducing its expression level in tumor cells to inhibit tumor adaptability and growth. In conclusion, through in-depth research and clinical application of the UBB gene, we can provide more precise and effective therapeutic options for ccRCC patients and significantly improve their prognosis.

## 5 Conclusion

This study highlights the importance of increased mitochondrial autophagy in ccRCC and its impact on tumor behavior. By advanced analysis, key genes such as TOMM7 and UBB were associated with autophagy and prognosis, with the role of UBB in ubiquitination emphasizing its therapeutic potential. These findings highlight the central role of mitochondrial autophagy in ccRCC, suggesting new therapeutic targets and improving personalized treatment for ccRCC patients.

## Data availability statement

The datasets presented in this study can be found in online repositories. The names of the repository/repositories and accession number(s) can be found in the article/supplementary material.

## Author contributions

LJ: Conceptualization, Data curation, Formal analysis, Visualization, Writing – original draft. XR: Conceptualization, Data curation, Writing – original draft. JY: Conceptualization, Data curation, Formal analysis, Writing – original draft. HQC: Formal analysis, Visualization, Writing – original draft. SZ: Formal analysis, Visualization, Writing – original draft. XZ: Formal analysis, Visualization, Writing – original draft. JH: Formal analysis, Visualization, Writing – original draft. CJ: Formal analysis, Visualization, Writing – original draft. YG: Formal analysis, Visualization, Writing – original draft. JT: Writing – review & editing. GY: Writing – review & editing, Conceptualization. HC: Writing – review & editing. JQ: Writing – review & editing.
